# Amniotic mesenchymal stromal cell-derived extracellular vesicles improve sperm motility in aged stallions

**DOI:** 10.3389/fvets.2026.1918660

**Published:** 2026-09-09

**Authors:** Anna Lange-Consiglio, Giulia Gaspari, Paola Gagni, Giampaolo Bosi, Monica Probo, Fausto Cremonesi, Federico Funghi

**Affiliations:** 1 Department of Veterinary Medicine and Animal Science (DIVAS), Università degli Studi di Milano, Milan, Italy; 2 Istituto di Scienze e Tecnologie Chimiche “Giulio Natta” (SCITEC), Consiglio Nazionale delle Ricerche (CNR), Milan, Italy; 3 Equine Veterinary Practitioner, Grosseto, Italy

**Keywords:** aged stallions, amniotic mesenchymal derived extracellular vesicles, equine reproductive medicine, sperm motility, stallion spermatozoa

## Abstract

**Introduction:**

Age-related testicular degeneration is a major cause of subfertility in stallions and is commonly associated with impaired sperm motility. Extracellular vesicles (EVs) derived from mesenchymal stromal/stem cells (MSCs) have recently emerged as promising regenerative mediators due to their ability to transfer bioactive molecules to target cells. This pilot study evaluated the effects of equine amniotic MSC-derived EVs (AMSC-EVs) on sperm motility and functional integrity in aged stallions.

**Methods:**

Semen samples collected from four aged stallions were analyzed by conventional spermiogram and computer-assisted sperm analysis (CASA). Amniotic MSC-EVs were isolated and characterized by nanoparticle tracking analysis, transmission electron microscopy, and Western blot analysis of EV markers. Following a dose–response assay, spermatozoa were co-incubated with PKH26-labelled AMSC-EVs, and sperm kinetic parameters, EV interaction, mitochondrial membrane potential, viability, and acrosomal status were assessed.

**Results:**

Co-incubation with AMSC-EVs at 400 × 10⁶ EVs/mL significantly modulated sperm kinetic parameters, attenuating the decline in progressive motility and maintaining higher linearity (LIN) and straightness (STR) values than untreated controls, while dynamically modifying the amplitude of lateral head displacement (ALH) (*p* < 0.05). Progressive motility was preserved up to 4 h post collection in EV-treated samples (16.00 ± 0.20%) compared with untreated controls (0.90 ± 0.10%). Fluorescence analyses by confocal microscopy confirmed EV localization predominantly at the sperm midpiece. EV-treated spermatozoa showed dynamic modulation of movement patterns without evidence of premature capacitation or acrosome reaction in viable spermatozoa.

**Discussion:**

These results suggest that AMSC-EVs may represent a promising cell-free strategy for modulating sperm functionality in aged stallions while preserving sperm physiological integrity. Considering the limited number of stallions included, the findings should be interpreted as preliminary proof-of-concept observations.

## Highlights

- AMSC-derived extracellular vesicles preserved sperm motility in aged stallions.
- Extracellular vesicles interacted with the sperm midpiece region.
- Extracellular vesicles treatment preserved progressive motility and maintained higher LIN and STR values than untreated controls.
- AMSC-EVs did not induce premature sperm capacitation or acrosome reaction.
- AMSC-EVs may represent a novel cell-free regenerative strategy in equine reproduction.

## Introduction

Age-related decline in reproductive efficiency represents a major challenge in equine breeding and reproductive medicine. In stallions, advancing age is frequently associated with progressive deterioration of semen quality, reduced sperm concentration, impaired sperm motility, and increased morphological abnormalities, ultimately leading to subfertility or infertility (1, 2). Among the reproductive alterations observed in aged stallions, idiopathic testicular degeneration, also referred to as age-related testicular degeneration, is one of the most common causes of declining fertility (1). Although the pathophysiology of reproductive aging remains incompletely understood, increasing evidence suggests that oxidative stress, mitochondrial dysfunction, impaired redox regulation, and chronic low-grade inflammation play pivotal roles in the progressive impairment of sperm function (2–4). Recent studies further demonstrated that stallion sperm metabolism is tightly regulated by mitochondrial substrate utilization and oxidative phosphorylation, highlighting the delicate balance between ATP production and reactive oxygen species generation required to preserve sperm functionality during aging and semen preservation (4, 5). Because equine spermatozoa strongly depend on mitochondrial oxidative phosphorylation for ATP production and flagellar movement, mitochondrial dysfunction may severely compromise sperm motility, membrane stability, and fertilizing ability (3).

Current therapeutic and management strategies aimed at preserving fertility in aging stallions remain limited. Hormonal therapies, antioxidant supplementation, semen processing techniques, and assisted reproductive technologies such as intracytoplasmic sperm injection (ICSI) may partially compensate for declining semen quality but do not directly reverse testicular degeneration (1). Therefore, novel regenerative and cell-free therapeutic approaches capable of improving sperm function without compromising fertilizing potential are of increasing interest in equine andrology.

Extracellular vesicles (EVs) are membrane-bound nanoparticles secreted by virtually all cell types and are now recognized as fundamental mediators of intercellular communication (6). EVs carry a complex cargo composed of proteins, lipids, mRNAs, microRNAs, metabolites, and signaling molecules capable of modulating the biological activity of recipient cells (7, 8). In recent years, mesenchymal stromal/stem cell-derived EVs (MSC-EVs) have attracted considerable attention in regenerative medicine because they reproduce many of the anti-inflammatory, immunomodulatory, antioxidant, and regenerative properties of their parental cells while avoiding several limitations associated with direct stem cell transplantation (9, 10). Due to these properties, MSC-EVs are increasingly considered promising cell-free therapeutic tools in several medical fields, including tissue repair, inflammatory diseases, and reproductive medicine (11–13).

Within the reproductive system, EVs play essential roles in gamete maturation, fertilization, embryo development, and embryo-maternal communication. Recent evidence highlighted the central role of EV-mediated communication in both male and female reproductive physiology, including sperm maturation, membrane remodeling, capacitation, motility regulation, and fertilization processes (14–16). Reproductive tract-derived EVs can interact with spermatozoa through the transfer of proteins, lipids, and regulatory RNAs capable of modulating sperm bioenergetics, membrane dynamics, and functional competence. Interestingly, several studies suggest that EVs preferentially localize within the sperm midpiece, a mitochondria-rich compartment essential for ATP production and flagellar activity, supporting the hypothesis that EV cargo may directly influence mitochondrial homeostasis and sperm motility persistence.

Among the different MSC sources, amniotic tissue represents a particularly attractive origin for EV production because of its accessibility, low immunogenicity, high proliferative potential, and strong anti-inflammatory properties. Previous studies from our group demonstrated that equine amniotic mesenchymal stromal cell-derived EVs (AMSC-EVs) possess significant regenerative and anti-inflammatory activity in several experimental models (17–19). Furthermore, AMSC-EVs contain regulatory microRNAs and proteins involved in inflammatory modulation, oxidative stress regulation, tissue repair, and cellular homeostasis (20–22). In equine reproduction, AMSC-EVs have already shown promising effects in the prevention of persistent post-breeding induced endometritis and in the modulation of reproductive inflammatory responses (23). In addition, endometrial and oviduct-derived EVs were previously shown to enhance equine sperm hyperactivation and improve *in vitro* fertilization outcomes, further supporting the biological relevance of EV-mediated reproductive communication (24).

Evidence from experimental models also suggests that AMSC-EVs may positively influence male reproductive function. As an indirect analogy, amniotic fluid-derived exosomes were reported to improve spermatogenesis and testicular recovery in a rat model of azoospermia (25). Although this *in vivo* model differs substantially from the present *in vitro* assessment of sperm kinetics, it suggests a broader biological potential of amniotic-derived EVs within the male reproductive system.

More recently, improving sperm metabolism and resistance to oxidative stress has emerged as a promising strategy for preserving sperm functionality during semen handling and assisted reproductive procedures (4, 26). In parallel, engineered EV-based approaches are increasingly being investigated as innovative modulators of sperm preservation and cryobiology (27). Considering the increasing interest in EV-based regenerative strategies for assisted reproductive technologies and reproductive biotechnologies (28), elucidating the effects of AMSC-EVs on sperm physiology may provide novel translational perspectives for equine reproductive medicine.

Therefore, the aim of the present pilot study was to evaluate the effects of AMSC-EVs on sperm motility in aged stallions. In addition, the study investigated whether EV supplementation could induce premature sperm capacitation or acrosome reaction, potentially compromising sperm fertilizing ability. We hypothesized that AMSC-EVs could improve sperm kinetic parameters through interaction with the sperm mitochondrial compartment while preserving sperm physiological integrity and acrosomal stability.

## Materials and methods

### Ethics statement

All veterinary procedures involving animals were conducted in accordance with relevant guidelines and regulations. The study was reported in accordance with the ARRIVE guidelines and complied with Directive 2010/63/EU on the protection of animals used for scientific purposes. Placental tissues were collected after physiological deliveries and did not involve invasive procedures or additional animal manipulation. The stallions included in the study were privately owned and routinely housed at the equine breeding facility where the study was conducted. Semen collection was performed according to routine reproductive management procedures approved for animal welfare. Written informed consent was obtained from all owners for the collection and experimental use of ejaculates and other biological samples for research purposes.

### Experimental design

The *in vitro* experimental study included the following sequential procedures:

(1) isolation of equine amniotic mesenchymal stromal cells (AMSCs) from term placentas; (2) collection of conditioned medium (CM), and isolation of AMSC-derived extracellular vesicles (AMSC-EVs); (3) characterization of EVs according to MISEV recommendations through nanoparticle tracking analysis (NTA), Western blot, and transmission electron microscopy (TEM); (4) fluorescent labeling of EVs with PKH26 dye; (5) evaluation of EV uptake by spermatozoa using confocal microscopy following a dose–response study; (6) assessment of sperm motility parameters by computer-assisted sperm analysis (CASA); and (7) evaluation of sperm viability, mitochondrial membrane potential, and acrosomal status using triple fluorescent staining.

**Figure 1:**
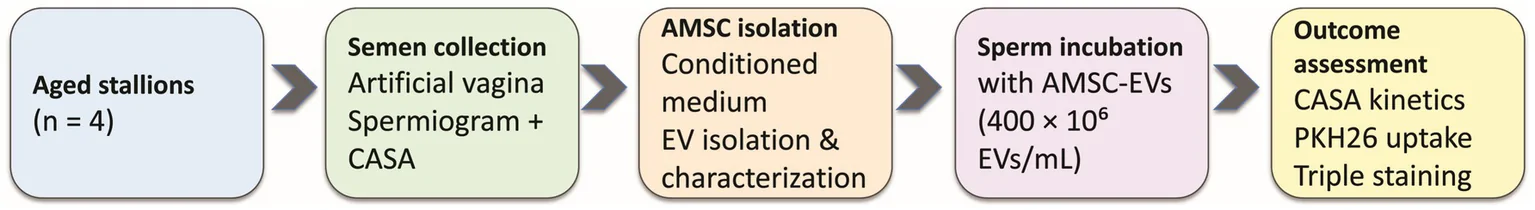
Schematic representation of the experimental workflow used to evaluate the effects of extracellular vesicles derived from equine amniotic mesenchymal stromal cells (AMSC-EVs) on sperm motility in aged stallions. Semen samples collected from aged stallions were incubated with AMSC-derived EVs (400 × 10^6^ EVs/mL), followed by CASA assessment of sperm kinetic parameters, evaluation of EV uptake by PKH26 fluorescence labeling, and analysis of sperm viability, mitochondrial membrane potential, and acrosomal status using triple fluorescent staining.

The experimental workflow of the study is summarized in Figure 1.

### Animals and semen collection

Four aged Trotter stallions (16–27 years old) were enrolled in the study during the 2024 breeding season. All stallions were housed at an equine clinic equipped with breeding facilities, where Dr. Funghi served as the sanitary director. Stallions had been routinely used for breeding throughout their reproductive life. According to their clinical and breeding history, a gradual deterioration of semen quality had been observed during aging, without a clearly identifiable onset. Complete and standardized historical reproductive indices were not available for all animals; therefore, the stallions were characterized based on their advanced age and impaired semen parameters rather than classified according to a quantitatively defined degree of subfertility.

Despite this decline, no clinical abnormalities of the reproductive tract were detected. Testicular palpation and ultrasonographic examination revealed normal testicular consistency and morphology in all animals.

Despite their advanced age, stallions were still included in breeding programs because of their previous athletic performance and reproductive value. Animals were maintained under standard management and feeding conditions, and no systemic diseases were reported at the time of semen collection.

Semen was collected using an artificial vagina according to routine reproductive management procedures. Only one ejaculate per stallion was included in the study because of the complexity and multi-step nature of the experimental design, which required extensive functional, imaging, and extracellular vesicle co-incubation analyses for each sample.

Although semen quality had progressively deteriorated during aging, the seminal characteristics of each stallion were considered relatively stable within the breeding period in which the experimental samples were collected.

Immediately after collection, semen samples underwent both macroscopic and microscopic evaluation. Macroscopic examination included assessment of semen volume and pH. Microscopic evaluation included sperm concentration, motility, morphology, and viability.

Sperm concentration was initially assessed using a Sperm-Count spectrophotometer (IMV Technologies, Italy) and subsequently confirmed using a Bürker counting chamber. Sperm motility was first evaluated by light microscopy and then further analyzed using a computer-assisted sperm analysis (CASA) system.

Sperm morphology was evaluated on stained smears by assessing both primary and secondary abnormalities using Rose Bengal and Victoria Blue staining. Briefly, sperm smears were stained with Rose Bengal for 10 min and Victoria Blue for 30 s, rinsed with distilled water, air-dried, and examined under light microscopy. Spermatozoa with intact acrosomes showed blue staining over the acrosomal region, whereas spermatozoa with acrosomal loss or damage exhibited uniform pink staining of the sperm head.

Sperm viability was evaluated using eosin-nigrosin staining. Briefly, semen was mixed with eosin–nigrosin stain, smeared onto glass slides, air-dried, and examined under light microscopy. Live spermatozoa remained unstained, whereas dead spermatozoa appeared pink/red against the dark background.

Fresh semen samples were diluted 1:1 (v:v) in pre-warmed INRA96 medium (IMV Technologies, Italy) and maintained at 38.5 °C in a humidified atmosphere containing 5% CO₂ until further processing. Samples were subsequently centrifuged at 100 × g for 1 min at 38.5 °C to remove debris and non-viable spermatozoa before extracellular vesicle co-incubation experiments.

### Isolation and culture of equine amniotic mesenchymal stromal cells

Three allanto-amniotic membranes were collected after spontaneous and physiological deliveries following the protocol of Lange-Consiglio et al. (29). Placental tissues were transported to the laboratory at 4 °C in sterile saline solution (0.9% NaCl) supplemented with 4 mg/mL amphotericin B, 100 U/mL penicillin, and 100 mg/mL streptomycin and processed within 8 h after collection.

The amniotic membrane was mechanically separated from the overlying allantois under sterile conditions and cut into smaller fragments of approximately 9 cm^2^, corresponding to a total tissue weight of approximately 12 g and an overall surface area of approximately 630 cm^2^. Tissue fragments were incubated in phosphate-buffered saline (PBS) containing 2.4 U/mL dispase for 9 min at 38.5 °C in order to facilitate epithelial detachment and extracellular matrix digestion. After incubation, fragments were transferred into high-glucose Dulbecco’s Modified Eagle Medium (HG-DMEM) supplemented with 10% fetal bovine serum (FBS) and 2 mM L-glutamine for 5–10 min at room temperature. Subsequently, amniotic fragments underwent enzymatic digestion with 1 mg/mL type I collagenase and 20 μg/mL DNase I for 3 h at 38.5 °C under gentle agitation. The resulting cell suspension was filtered through sterile cell strainers to remove undigested tissue debris and centrifuged at 300 × g for 10 min. Cell pellets were resuspended in complete HG-DMEM supplemented with 10% FBS, 100 U/mL penicillin, 100 mg/mL streptomycin, and 2 mM L-glutamine.

Cells were seeded in tissue culture flasks and incubated at 38.5 °C in a humidified atmosphere containing 5% CO₂. Non-adherent cells were removed after 24 h by medium replacement, whereas adherent cells displaying fibroblast-like morphology were maintained in culture until reaching approximately 80–90% confluence. Culture medium was replaced every 2–3 days. Cells at passage 3 were used for conditioned medium production and EV isolation.

### Conditioned medium production and EV isolation

At passage 3, AMSCs were cultured overnight in serum-free HG-DMEM to allow collection of conditioned medium (CM). The CM was collected and subjected to sequential centrifugation steps in order to remove cells and cellular debris before EV isolation.

Extracellular vesicles were isolated by ultracentrifugation according to previously described protocols and in agreement with MISEV guidelines (7, 8). Briefly, conditioned medium was centrifuged at low speed to eliminate residual cells and apoptotic bodies, followed by ultracentrifugation at 100,000 × g for EV pelleting. The resulting EV pellet was washed in sterile PBS and ultracentrifuged again under the same conditions to reduce protein contaminants. Final EV pellets were resuspended in sterile PBS and stored at −80 °C until use.

### Extracellular vesicle characterization

Extracellular vesicles were characterized according to their size distribution and concentration by nanoparticle tracking analysis, protein marker expression by Western Blot, and morphology by transmission electron microscopy

- Nanoparticle tracking analysis

AMSC-EV size distribution and concentration were evaluated by nanoparticle tracking analysis (NTA) using a NanoSight NS300 system (Malvern Technologies, Malvern, UK) equipped with a 532 nm laser. Samples were diluted in filtered PBS to a final volume of 1 mL. Several dilutions were tested to obtain an optimal particle concentration, corresponding to approximately 20–100 particles per frame. After NTA analysis, EV aliquots were stored at −80 °C until subsequent analyses.

- Transmission electron microscopy

Transmission electron microscopy (TEM) was performed for ultrastructural characterization of EVs and spermatozoa. Briefly, 10 μL of EV suspension at a concentration of 20 × 10^8^ particles/mL were adsorbed onto 300-mesh formvar/carbon-coated copper grids. Samples were fixed with 2.5% glutaraldehyde for 5 min and repeatedly washed with distilled water. Grids were negatively stained with 2% uranyl acetate, air dried, and examined using a transmission electron microscope as previously described. Digital images were acquired for morphological analysis.

- Western blot analysis

EV characterization was performed by Western blot analysis to detect canonical EV markers. To reduce technical and biological variability, and due to limited sample availability, AMSC-EV samples were pooled. Briefly, 30ug of total protein was prepared under reducing conditions in Laemmli buffer, heated at 95 °C for 10 min, separated by SDS-PAGE on 4–20% TGX Precast Protein Gels (Bio-Rad), and transferred onto nitrocellulose membranes using the Trans-Blot Turbo system (Bio-Rad, Milan, Italy). Membranes were blocked for 1 h in 5% BSA in TBS-T and incubated overnight at 4 °C with primary antibodies against TSG101 (Invitrogen, 1:500), and Alix (Santa Cruz, 1:1,000). Signals were detected using Clarity Western ECL Substrate (Bio-Rad) and acquired with a ChemiDoc XRS + system (Bio-Rad).

To evaluate EV purity, comparative protein quantification and Western blot analyses were performed on EVs and AMCs using Calnexin as a negative marker for cellular contamination. AMC pellets were lysed in RIPA buffer (Thermo Fisher) supplemented with Halt Protease Inhibitor (1×), while EV suspensions were mixed 1:1 with the same lysis buffer. Samples were sonicated, centrifuged at 14,000 × g for 15 min, and protein concentration was determined by BCA assay (Pierce™). Protein extracts were normalized to equal loading (30 μg per lane), separated on 4–15% TGX gradient gels (Bio-Rad), and transferred as described above. Membranes were incubated with anti-Calnexin primary antibody (Sigma, 1:1,000), followed by HRP-conjugated goat anti-mouse secondary antibody (Jackson ImmunoResearch, 1:2,500 in 1% BSA/TBS-T). Chemiluminescent detection was performed using ECL substrate (Bio-Rad) and imaged with a ChemiDoc™ SRX system (Bio-Rad).

### Fluorescent labeling of extracellular vesicles and evaluation of EV uptake by spermatozoa

Extracellular vesicles were fluorescently labeled using PKH26, a red aliphatic fluorochrome that intercalates into phospholipid bilayers. Briefly, isolated EVs were ultracentrifuged, and the resulting pellet was resuspended in 1 mL of Diluent C provided with the PKH26 kit. Four microliters of PKH26 fluorochrome were added, and samples were incubated for 30 min at 38.5 °C in a humidified atmosphere containing 5% CO₂ under dark conditions. The labeling reaction was stopped by adding 7 mL of serum-free DMEM. Samples were ultracentrifuged again at 100,000 × g for 1 h at 4 °C to remove unbound dye. The final EV pellet was resuspended in HG-DMEM and stored at −20 °C until use. The nominal concentration of labelled EVs used for sperm co-incubation was calculated from the initial NTA-derived stock concentration and the final resuspension volume. EV concentration was not re-measured by NTA after PKH26 labelling and the additional ultracentrifugation step; therefore, some particle loss during processing cannot be excluded.

Before sperm co-incubation, PKH26-labelled EVs were examined alone by fluorescence microscopy to confirm successful labelling. Unlabeled spermatozoa were used as a negative fluorescence control. A dye-only control processed in the absence of EVs was not performed.

A dose–response study previously performed by our group evaluated the effects of AMSC-EV concentration ranging from 50 to 500 × 10^6^ EVs/mL on equine sperm kinetic parameters during a 4-h post-collection monitoring period (23). Based on these results, a concentration of 400 × 10^6^ EVs/mL was selected for the present experiments because it provided the most favorable overall preservation of sperm kinetic parameters.

For EV uptake analysis, spermatozoa were co-incubated with PKH26-labelled AMSC-EVs at a concentration of 400 × 10^6^ EVs/mL. Sperm nuclei were counterstained with Hoechst 33342. Following co-incubation, samples were analyzed by confocal laser scanning microscopy. Positive control staining was performed on EVs alone and spermatozoa alone using PKH-26 and Hoechst, respectively.

### Computer-assisted sperm analysis (CASA)

Sperm motility parameters were evaluated using a computer-assisted sperm analysis (CASA) system immediately after semen collection (CTR) and at 1, 2, 3, and 4 h post-collection. Each ejaculate was divided into matched control and EV-treated aliquots, which were incubated in parallel.

A customized CASA system was assembled with an Olympus BX51 microscope fitted with a warming stage, negative-phase contrast optics (20× objective and 10× ocular) and a Basler (model A6021–2; Sintak S.r.l., Corsico, Mi, Italy) video camera interfaced with a computer to digitize and analyze the image. The software used for image acquisition and analysis was Image-Pro Plus 5.1-Media Cybernetics (Immagini and Computer, Bareggio, Milano, Italy). An aliquot of 5 μL of diluted semen was pipetted into a prewarmed 20-μm depth counting chamber (Cell-Vu Chambers; Fertility Technologies Inc., IMV, Piacenza, Italy). Twenty frames were analyzed at a sampling frequency of 30 frames/s. The threshold velocity was set at 8 μm/s and the maximum velocity at 150 μm/s. The minimum numbers of sampling points were 1 for motility, 3 for velocity, and 7 for ALH calculation. The pixel scale was 0.688 μm/pixel, and the sperm cell-size range was set at 4–15 pixels.

CASA evaluation included analysis of total motility, progressive motility, curvilinear velocity (VCL), straight-line velocity (VSL), average path velocity (VAP), amplitude of lateral head displacement (ALH), linearity (LIN), and straightness (STR). VCL was defined as the average velocity measured along the actual curvilinear trajectory of the sperm cell, whereas VSL represented the average velocity measured along the straight line connecting the initial and final sperm positions. VAP represented the average velocity calculated along the smoothed average sperm path. ALH corresponded to the amplitude of lateral displacement of the sperm head from its average trajectory. Linearity (LIN) was calculated as the ratio between VSL and VCL.

For each stallion, treatment condition, and incubation time, 10 microscopic fields and at least 200 spermatozoa were evaluated. The measurements obtained from the 10 fields were averaged to generate a single biological value for each stallion. Microscopic fields and individual spermatozoa were considered technical observations and were not treated as independent experimental replicates.

All analyses were performed under standardized environmental and optical conditions to minimize analytical variability.

Following the dose–response study, sperm samples were co-incubated with AMSC-EVs at a concentration of 400 × 10^6^ EVs/mL, which was identified as the concentration producing the most evident favorable overall preservation of sperm kinetic parameters. Semen aliquots were maintained at 38.5 °C throughout the experiment and analyzed at the specific post-collection time points.

### Triple fluorescent staining

Triple fluorescent staining was performed to simultaneously evaluate sperm viability, mitochondrial membrane potential, and acrosomal status following incubation with AMSC-derived EVs. Triple fluorescent staining was performed as previously described by de Andrade et al. (30), with minor modifications.

The staining protocol included propidium iodide (PI), 5,5′,6,6′-tetrachloro-1,1′,3,3′-tetraethylbenzimidazolylcarbocyanine iodide (JC-1), and fluorescein isothiocyanate-conjugated peanut agglutinin (FITC-PNA). The PI was used to assess plasma membrane integrity and identify non-viable spermatozoa, whereas JC-1 staining was used to evaluate mitochondrial membrane potential. Staining with FITC-PNA was used to assess acrosomal membrane integrity and detect acrosome-reacted spermatozoa. Following co-incubation with EVs, sperm samples were stained according to the established protocol and analyzed by fluorescence microscopy. At least 200 spermatozoa per sample were evaluated in three technique replicates. The interpretation of fluorescence patterns and sperm classification following triple staining were performed in a blinded manner by an experienced operator to minimize observer-related bias.

Spermatozoa were classified according to the fluorescence patterns generated by the combined staining procedure and categorized as viable, dead, transitional, or acrosome-reacted spermatozoa. Viable spermatozoa with high mitochondrial membrane potential were identified by intact plasma membranes and red JC-1 mitochondrial fluorescence, whereas spermatozoa undergoing acrosome reaction were identified by FITC-PNA-positive fluorescence in the acrosomal region. Attention was given to the identification of viable spermatozoa undergoing premature acrosome reaction following EV exposure. The interpretation of fluorescence patterns used for sperm classification is summarized in Table 1.

**Table 1:** Interpretation of fluorescence patterns obtained by triple staining with PI, JC-1, and FITC-PNA.

Fluorescence pattern	Interpretation	Functional status
Colorless head/Red mitochondria (TI/MR)	Viable spermatozoa with intact plasma membrane and high mitochondrial membrane potential	Viable
Green colorless head/Red mitochondria (TIV/MR)	Viable spermatozoa with high mitochondrial membrane potential and acrosome reaction	Viable reacted
Colorless head/Green mitochondria (TI/MV)	Viable spermatozoa with low mitochondrial activity	Transitional
Green colorless head/Green mitochondria (TIV/MV)	Spermatozoa with low mitochondrial activity and acrosome reaction	Hypothetical transitional pattern
Red head/Red mitochondria (TR/MR)	Spermatozoa with destabilized plasma membrane and preserved mitochondrial membrane potential	Transitional
Red-green head/Red mitochondria (TRV/MR)	Spermatozoa with destabilized plasma membrane, preserved mitochondrial membrane potential, and acrosome reaction	Transitional reacted
Red head/Green mitochondria (TR/MV)	Dead spermatozoa with low mitochondrial membrane potential	Dead
Red-green head/Green mitochondria (TRV/MV)	Dead spermatozoa with low mitochondrial membrane potential and acrosome reaction	Dead reacted

### Statistical analysis

All data are presented as mean ± standard deviation (SD). Statistical analyses were performed using GraphPad Prism software (GraphPad Software Inc., San Diego, CA, USA).

The stallion was considered the experimental unit, with four independent biological replicates (*n* = 4). For each stallion, treatment condition, and incubation time, the CASA measurements obtained from 10 microscopic fields and at least 200 spermatozoa were averaged to generate a single biological value. Microscopic fields and individual spermatozoa were considered technical observations and were not treated as independent experimental replicates. Control and EV-treated data were paired within each stallion.

Sperm motility parameters obtained by CASA analysis, as well as fluorescence data related to sperm viability, mitochondrial membrane potential, and acrosomal status, were analyzed using two-way repeated measures analysis of variance (ANOVA), considering treatment (CTR vs. EV) and post-collection time (0, 1, 2, 3, and 4 h) as main factors. Multiple comparisons were performed using Tukey’s *post-hoc* test.

For fluorescence analyses, at least 200 spermatozoa were evaluated per slide, and three slides were analyzed for each experimental condition. The values obtained from the three slides were averaged to generate a single value for each stallion, treatment condition, and incubation time. The slides and individual spermatozoa were considered technical observations and were not treated as independent experimental replicates.

Differences were considered statistically significant at *p* ≤ 0.05. Given the limited number of stallions, the statistical findings were interpreted within the preliminary proof-of-concept nature of the study.

## Results

### Isolation and expansion of equine amniotic mesenchymal stromal cells

Equine amniotic mesenchymal stromal cells (AMSCs) were successfully isolated from amniotic membrane samples collected after parturition. Cells displayed fibroblast-like morphology and plastic adherence typical of mesenchymal stromal cells throughout culture expansion. The isolation procedure was reproducible, and no contamination events were observed during culture maintenance. AMSCs at passage three were used for conditioned medium production and EV isolation.

### Extracellular vesicle characterization

**Figure 2:**
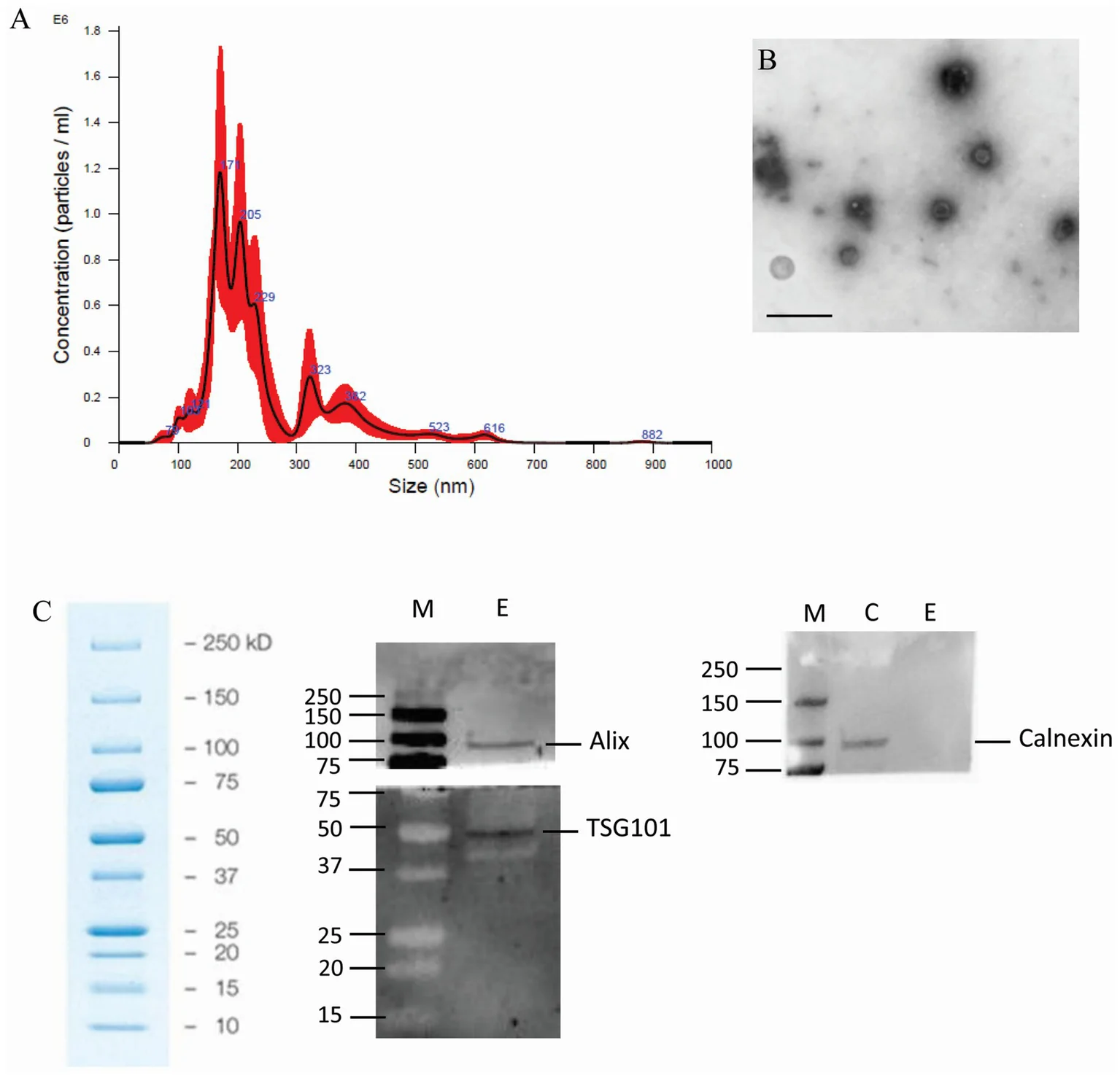
Characterization of AMSC-derived EVs. **(A)** Nanoparticle tracking analysis showing EV size distribution and concentration **(B)** Transmission electron microscopy image showing spherical electron-dense EVs. **(C)** Western blot analyses confirming the expression of EV-associated markers Alix, TSG101, and negativity for Calnexin.

Nanoparticle tracking analysis (NTA) revealed a heterogeneous EV population with a mean particle diameter of 243.3 ± 10.8 nm and a concentration of 1.14 × 10^10^ ± 1.73 × 10^9^ particles/mL. Given the broad size distribution, the preparation was not assigned as to a specific EV size subclass (Figure 2A).

Transmission electron microscopy (TEM) confirmed the efficiency of the EV isolation protocol, revealing spherical electron-dense vesicles with morphology consistent with EVs (Figure 2B).

Western blot analysis confirmed the expression of the EV-associated intracellular markers Alix and TSG101, further confirming the presence of EVs within the isolated preparation (Figure 2C). Negativity to Calnexin confirmed absence of cells.

### Baseline semen characteristics of aged stallions

Baseline semen evaluation confirmed age-related impairment of seminal quality in all enrolled stallions (Table 2). Compared with reproductively efficient adult stallions, which typically show sperm motility >60–70%, sperm concentrations frequently exceeding 100 × 10^6^ spermatozoa/mL, and lower percentages of morphological abnormalities, the enrolled aged stallions showed reduced motility, low sperm concentration, and increased secondary abnormalities, consistent with age-related testicular degeneration (1). Sperm viability remained moderately preserved despite the reduced kinetic performance, indicating that impaired sperm functionality in aged stallions may not exclusively depend on loss of membrane integrity but could also involve subcellular dysfunctions affecting sperm motility and bioenergetic competence.

**Table 2:** Semen characteristics of aged stallions included in the study.

Stallion	Volume (mL)	pH	Motile spermatozoa (%)	Concentration (million/mL)	Primary abnormalities (%)	Secondary abnormalities (%)	Viability (%)
C1 (16 years)	80	7.6	47.50	11.01	6.83	27.34	67.60
C2 (18 years)	120	7.8	30.30	33.57	8.74	39.79	75.00
C3 (27 years)	95	7.8	57.40	18.65	9.27	27.15	57.60
C4 (23 years)	110	7.6	46.20	17.89	4.47	26.78	64.66
Mean ± SD	101.25 ± 17.50	7.7 ± 0.12	45.35 ± 11.21	20.28 ± 9.50	7.33 ± 2.17	30.27 ± 6.35	66.22 ± 7.20

Values are reported as mean ± standard deviation (SD). Microscopic evaluation included assessment of sperm motility, sperm concentration, primary and secondary morphological abnormalities, and sperm viability.

### Interaction between spermatozoa and EVs

**Figure 3:**
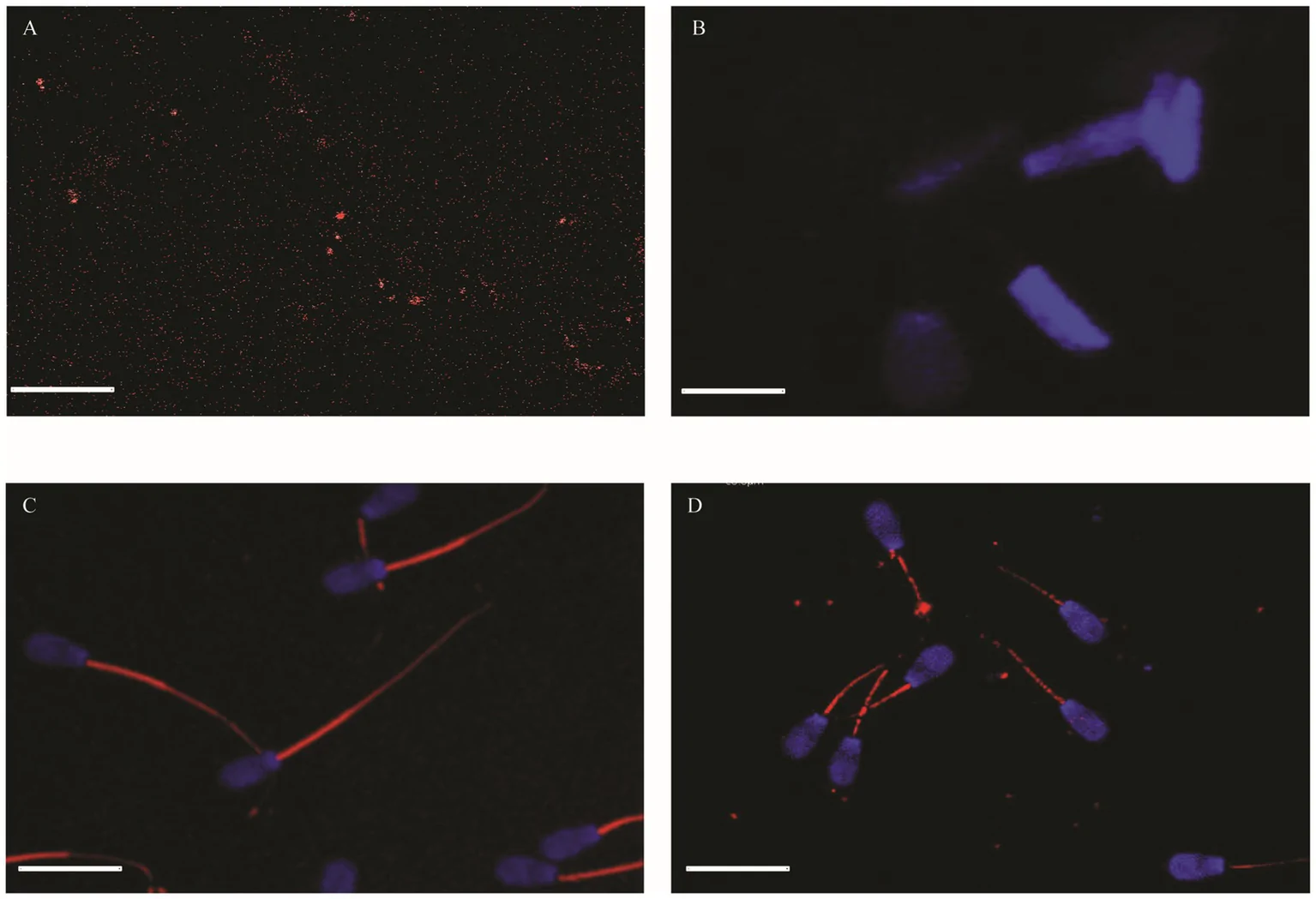
Uptake of AMSC-derived EVs by spermatozoa. **(A)** PKH26-labelled extracellular vesicles showing positive red fluorescence before sperm co-incubation. **(B)** Hoechst-labelled spermatozoa showing blue nuclear fluorescence in the absence of EV staining. **(C)** Homogeneous localization and distribution of PKH26-labelled EVs along spermatozoa following co-incubation with 400 × 10^6^ EVs/mL. **(D)** Less uniform EV association observed at lower EV concentrations, characterized by scattered fluorescent aggregates. Red fluorescence: PKH26-labelled EVs; blue fluorescence: Hoechst-labelled sperm nuclei.

Fluorescence analysis demonstrated stable EV-associated fluorescence throughout the 4 h incubation period, indicating sustained association between EVs and spermatozoa. PKH26-labelled AMSC-derived EVs showed intense and homogeneous red fluorescence before co-incubation (Figure 3A), whereas Hoechst staining allowed visualization of sperm nuclei in the absence of EV fluorescence (Figure 3B).

Following co-incubation, AMSC-derived EVs were efficiently localized in spermatozoa (Figures 3C,D). The preliminary dose–response study revealed that the concentration of 400 × 10^6^ EVs/mL resulted in the most homogeneous EV distribution along spermatozoa and was associated with the best improvement in sperm kinetic parameters (Figure 3C). In contrast, lower EV concentrations resulted in less uniform fluorescence patterns characterized by scattered fluorescent aggregates (Figure 3D).

Fluorescence signals were predominantly localized at the level of the sperm midpiece and proximal flagellum, supporting preferential interaction of EVs with mitochondria-rich sperm compartments.

### Effects of AMSC-EVs on sperm motility

**Figure 4:**
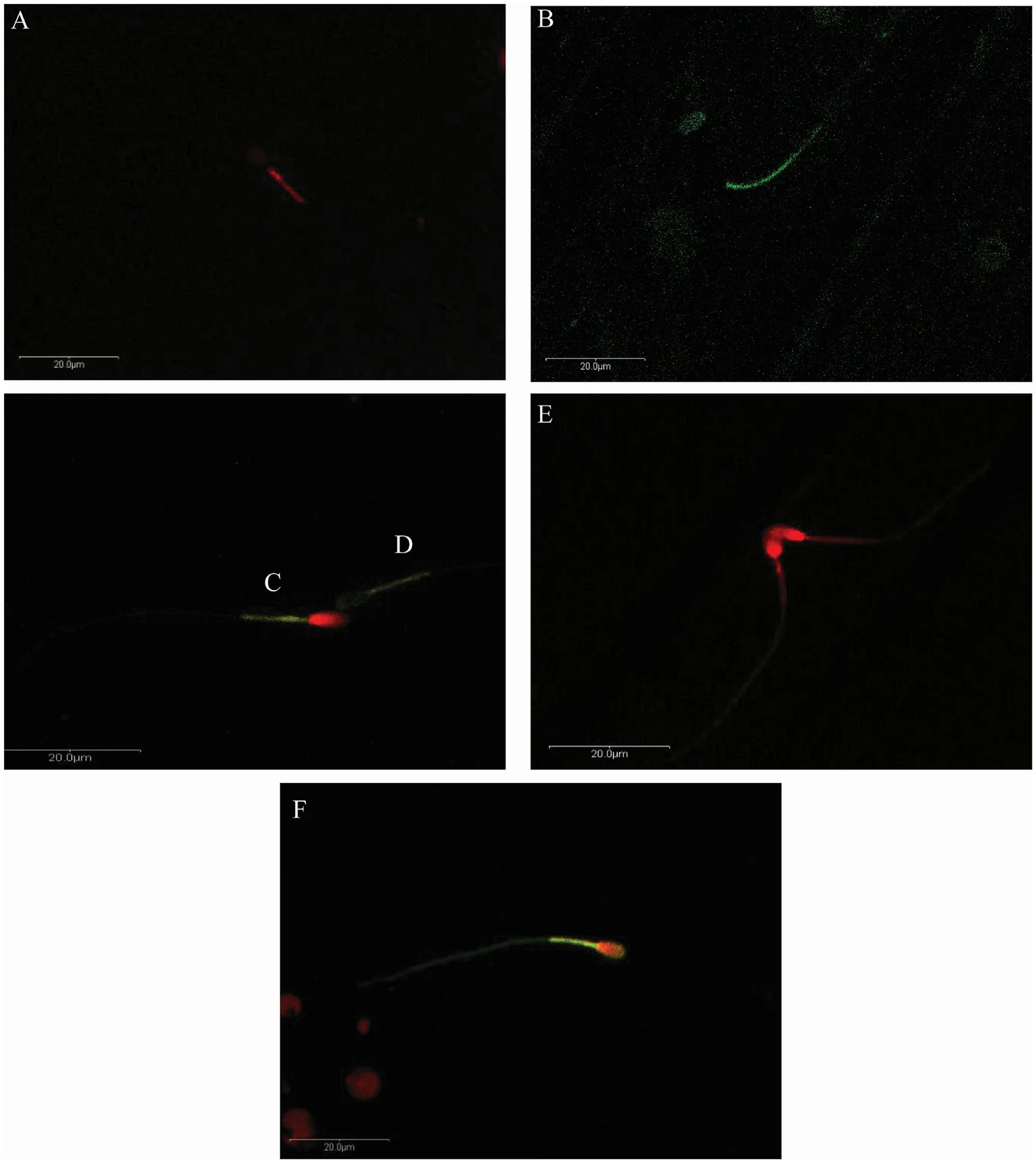
Representative fluorescence patterns of spermatozoa evaluated by combining viability, acrosomal status, and mitochondrial membrane potential staining. **(A)** Live spermatozoon with an intact plasma membrane (unstained head) and high mitochondrial membrane potential (red midpiece fluorescence). **(B)** Viable acrosome-reacted spermatozoon with low mitochondrial membrane potential (green midpiece fluorescence). **(C)** Dead spermatozoon showing compromised plasma membrane integrity (red head fluorescence) and low mitochondrial membrane potential. **(D)** Transitional spermatozoon with reduced mitochondrial membrane potential. **(E)** Transitional spermatozoa with low mitochondrial membrane potential. **(F)** Dead acrosome-reacted spermatozoon with low mitochondrial membrane potential. Scale bar: 20 μm.

Co-incubation with AMSC-derived EVs significantly influenced several sperm kinetic parameters during the 4-h post collection monitoring period (Table 3 and Figure 4).

**Table 3:** (A) Effect of EVs on sperm motility percentages during the 4-h post-collection monitored period. (B) Effect of EVs on sperm kinetic parameters during the 4-h post-collection monitored period. (C) Effect of EVs on ALH, LIN, and STR parameters during the 4-h post collection monitoring period.

A				
Time	CTR motile (%)	EV motile (%)	CTR progressive (%)	EV progressive (%)
0 h	47.78 ± 1.13	—	22.54 ± 1.63	—
1 h	38.10 ± 2.34a	34.40 ± 3.28a	13.62 ± 2.54a	17.32 ± 2.12aA
2 h	29.18 ± 2.94b	30.06 ± 1.12b	7.56 ± 4.99b	19.18 ± 3.62aA
3 h	28.26 ± 1.96b	27.34 ± 1.46c	3.92 ± 3.35c	19.28 ± 1.86aA
4 h	23.91 ± 1.22c	25.29 ± 1.62d	0.90 ± 0.10d	16.00 ± 0.20bA

B						
Time	CTR VCL (μm/s)	EV VCL (μm/s)	CTR VSL (μm/s)	EV VSL (μm/s)	CTR VAP (μm/s)	EV VAP (μm/s)
0 h	96.33 ± 8.43	—	45.61 ± 1.96	—	84.71 ± 2.60	—
1 h	83.97 ± 3.34a	79.52 ± 2.51aA	36.34 ± 1.69a	34.18 ± 2.71a	74.47 ± 2.80a	76.35 ± 1.90a
2 h	76.88 ± 2.27b	89.29 ± 2.19bA	30.38 ± 2.54b	32.85 ± 2.30a	70.47 ± 1.12b	77.93 ± 1.06aA
3 h	78.68 ± 1.34b	88.71 ± 1.43bA	27.03 ± 1.92c	32.02 ± 1.47aA	71.71 ± 1.34b	78.02 ± 3.33aA
4 h	78.83 ± 2.45b	80.18 ± 1.58a	27.93 ± 1.89c	29.46 ± 1.89b	68.93 ± 2.34c	78.28 ± 1.57aA

C						
Time	CTR ALH (μm)	EV ALH (μm)	CTR LIN (%)	EV LIN (%)	CTR STR	EV STR
0 h	4.68 ± 0.77	—	44.89 ± 1.85	—	0.73 ± 0.28	—
1 h	4.20 ± 0.91	4.15 ± 0.66	38.55 ± 2.30a	49.04 ± 2.40aA	0.73 ± 0.38	0.76 ± 0.26aA
2 h	4.92 ± 0.69	3.50 ± 0.32aA	37.79 ± 2.51a	49.43 ± 1.98aA	0.73 ± 0.36	0.78 ± 0.23bA
3 h	4.58 ± 0.64	4.10 ± 0.66aA	37.41 ± 1.18a	49.28 ± 2.09aA	0.69 ± 0.26a	0.76 ± 0.28aA
4 h	4.13 ± 0.51	5.72 ± 0.19bA	31.16 ± 1.51b	44.87 ± 2.75bA	0.62 ± 0.20b	0.75 ± 0.39aA

Values are expressed as mean ± standard deviation (SD) with *n* = 4 stallions. The 0-h value represents the common baseline measured before EV supplementation and is therefore reported only once. Different lowercase superscript letters indicate statistically significant differences among incubation times within the same experimental condition (*p* ≤ 0.05). Uppercase superscript letters indicate statistically significant differences between control and EV-treated at the same time point (*p* ≤ 0.05). No control-versus-EV comparison was performed at 0 h.

Although total sperm motility progressively declined over time in both experimental groups, EV-treated spermatozoa maintained significantly higher progressive motility than untreated controls from 1 to 4 h post collection (*p* ≤ 0.05) (Tables 3A–C).

In the control group, progressive motility decreased from 22.54 ± 1.63% at baseline to 0.90 ± 0.10% at 4 h post-collection, whereas EV-treated samples maintained higher progressive motility values, reaching 16.00 ± 0.20% at 4 h (*p* ≤ 0.05). Significant differences between EV-treated and untreated spermatozoa were already evident after 1 h of incubation (17.32 ± 2.12% vs. 13.62 ± 2.54%, respectively, *p* ≤ 0.05) and became progressively more pronounced at later time points.

EV supplementation also significantly influenced sperm velocity parameters (Table 3B). In untreated samples, curvilinear velocity (VCL), straight-line velocity (VSL), and average path velocity (VAP) progressively decreased during incubation. In contrast, EV-treated spermatozoa maintained significantly higher VCL and VAP values, particularly at 2 h and 3 h (*p* ≤ 0.05). At 2 h, VCL values reached 89.29 ± 2.19 μm/s in EV-treated spermatozoa compared with 76.88 ± 2.27 μm/s in controls, whereas VAP values were 77.93 ± 1.06 μm/s and 70.47 ± 1.12 μm/s, respectively (*p* ≤ 0.05). Similarly, VSL values remained significantly higher in EV-treated spermatozoa at 3 h (32.02 ± 1.47 μm/s) compared with untreated controls (27.03 ± 1.92 μm/s, *p* ≤ 0.05), indicating better maintenance of directional sperm movement during prolonged incubation.

Among the evaluated CASA parameters, the most evident EV-associated effects were observed for linearity (LIN), straightness (STR), and amplitude of lateral head displacement (ALH) (Table 3C). EV-treated spermatozoa maintained significantly higher LIN values throughout incubation compared with controls. At 4 h, LIN values were 44.87 ± 2.75% in EV-treated samples versus 31.16 ± 1.51% in untreated spermatozoa (*p* ≤ 0.05). Similarly, STR values remained significantly higher in the EV group throughout the incubation period, reaching 0.75 ± 0.39 at 4 h compared with 0.62 ± 0.20 in controls (*p* ≤ 0.05).

EV supplementation also significantly modified ALH values over time. EV-treated spermatozoa showed lower ALH values than controls at 2 h and 3 h (*p* ≤ 0.05), whereas a significant increase was observed at 4 h (5.72 ± 0.19 μm in EV-treated samples vs. 4.13 ± 0.51 μm in controls, *p* ≤ 0.05). Overall, these findings suggest that AMSC-EVs preserved sperm progressive motility while dynamically modulating sperm movement patterns during prolonged incubation (see Table 4).

**Table 4:** Effect of EVs on sperm viability and acrosomal status evaluated by triple staining during the 4-h post-collection monitoring period.

Group	Live [%]	Dead [%]	Transitional [%]	Dead + RA [%]
CTR 0 h	51.12 ± 1.32ᵃ	39.44 ± 2.12ᵃ	8.44 ± 0.44ᵃ	1.00 ± 0.71ᵃ
CTR 1 h	40.40 ± 3.11ᵇ	45.44 ± 2.33ᵇ	6.20 ± 0.45ᵇ	7.96 ± 0.54ᵇ
CTR 2 h	35.12 ± 2.31ᶜ	49.31 ± 3.58ᶜ	12.45 ± 1.22ᶜ	3.12 ± 2.41ᶜ
CTR 3 h	34.44 ± 2.62ᶜ	51.32 ± 3.48ᶜ	12.22 ± 1.26ᶜ	2.02 ± 0.37ᶜ
CTR 4 h	29.66 ± 2.84ᵈ	52.27 ± 3.26ᵈ	15.63 ± 1.44ᵈ	2.44 ± 0.42ᶜ
EVs 1 h	39.76 ± 2.13ᵇ	46.82 ± 3.65ᵇ	5.00 ± 0.45ᵇ	8.42 ± 0.61ᵇ
EVs 2 h	35.74 ± 2.39ᶜ	48.12 ± 3.81ᶜ	14.27 ± 1.22ᶜ	1.87 ± 1.32ᶜ
EVs 3 h	35.22 ± 3.11ᶜ	50.22 ± 3.29ᶜ	13.56 ± 1.95ᶜ	1.00 ± 0.21ᶜ
EVs 4 h	31.12 ± 1.55ᵈ	53.78 ± 3.22ᵈ	13.43 ± 1.39ᶜ	1.67 ± 0.21ᶜ

Values are expressed as mean ± SD, with *n* = 4 stallions. The 0-h values represent the common baseline measured before EV supplementation and are therefore reported only once. They do not represent a separate control and EV-treated baseline assessment. Spermatozoa were classified by triple staining into live, dead, transitional, and dead acrosome-reacted (dead + RA) cells. Evaluations were performed at 0, 1, 2, 3, and 4 h post-collection. CTR, control group; EV, extracellular vesicle-treated group; RA, acrosome reaction. Lowercase superscript letters indicate significant differences over time within the same experimental group (*p* < 0.05).

### Triple fluorescent staining

Transitional spermatozoa moderately increased during incubation in both groups, reaching 15.63 ± 1.44% in controls and 13.43 ± 1.39% in EV-treated samples at 4 h. Dead acrosome-reacted spermatozoa remained low throughout incubation, with values ranging from 1.00 ± 0.71% at baseline to 2.44 ± 0.42% at 4 h in controls and from 8.42 ± 0.61% at 1 h to 1.67 ± 0.21% at 4 h in EV-treated spermatozoa. At 1 h, the percentage of dead acrosome-reacted spermatozoa in the EV-treated group (8.42 ± 0.61%) was comparable to that observed in the control group (7.96 ± 0.54%), with no statistically significant difference between treatments. This transient increase was restricted to non-viable spermatozoa and was not maintained at subsequent incubation times.

No statistically significant differences were observed between EV-treated and control groups for live, dead, transitional, or dead acrosome-reacted sperm populations at corresponding incubation times (*p* > 0.05). Importantly, EV supplementation did not induce premature acrosome reaction in viable spermatozoa, supporting preservation of sperm physiological integrity during *in vitro* incubation. Fluorescence analyses demonstrated that acrosomal alterations were predominantly associated with spermatozoa exhibiting compromised plasma membrane integrity and reduced mitochondrial membrane potential rather than with viable sperm cells.

Overall, triple staining analysis demonstrated that AMSC-derived EV supplementation preserved sperm kinetic behavior without inducing significant alterations in sperm viability, mitochondrial membrane integrity, or acrosomal stability during the 4-h post-collection monitoring period. These findings support the hypothesis that EV treatment enhances sperm functionality while preserving sperm physiological competence.

Despite the better preservation of sperm kinetic activity observed after EV supplementation, fluorescence analysis demonstrated no evidence of premature acrosome reaction in viable spermatozoa. In particular, viable sperm cells showing high mitochondrial membrane potential were not associated with FITC-PNA-positive acrosomal staining, indicating preservation of acrosomal membrane integrity. Most spermatozoa exhibiting acrosome reaction were characterized by compromised plasma membrane integrity and reduced mitochondrial membrane potential, indicating that acrosomal alterations mainly occurred in non-viable or degenerating sperm cells rather than as a consequence of EV exposure.

These findings suggest that the dynamic modulation of sperm motility induced by AMSC-derived EVs was not associated with premature sperm capacitation and therefore did not appear to compromise sperm physiological competence.

## Discussion

The present pilot study investigated the effects of AMSC-EVs on sperm functionality in aged stallions characterized by progressive age-related seminal impairment and declining fertility, as demonstrated by reduced sperm motility, low sperm concentration, and increased secondary morphological abnormalities. Such age-associated reproductive impairment is commonly linked to progressive deterioration of spermiogram parameters secondary to age-related testicular degeneration and mitochondrial dysfunction (1, 3). Within this clinical context, the present study demonstrated that AMSC-EVs significantly modulated sperm kinetic parameters without evidence of premature acrosome reaction in viable spermatozoa. Treatment with AMSC-EVs supported progressive motility, linearity (LIN), and straightness (STR), indicating preserved maintenance of directional sperm movement during the 4-h post-collection monitoring period. In addition, AMSC-EV-treated spermatozoa showed dynamic changes in amplitude of lateral head displacement (ALH), characterized by lower values during the intermediate incubation period followed by a significant increase at 4 h. This pattern suggests that AMSC-EVs may not simply preserve sperm motility but rather modulate sperm movement dynamics over time. Fluorescence analyses further demonstrated AMCs-EV interaction within spermatozoa, predominantly at the level of the sperm midpiece, supporting the hypothesis of a direct interaction between EV cargo and sperm bioenergetic compartments, as previously observed in equine reproductive models (23, 24). Collectively, these findings suggest that AMSC-EVs may represent a promising cell-free strategy for improving sperm functionality in aging stallions.

Age-related reproductive decline is frequently associated with reduced semen quality, impaired sperm motility, and increased morphological abnormalities in stallions affected by age-related testicular degeneration (1). In agreement with previous reports, the stallions enrolled in the present study showed reduced sperm concentration, decreased progressive motility, and increased secondary abnormalities, confirming the presence of age-associated seminal impairment. Since equine spermatozoa strongly depend on mitochondrial oxidative phosphorylation for ATP production and flagellar activity, mitochondrial dysfunction and oxidative stress are considered major contributors to reduced sperm functionality during aging (3–5). Recent evidence also highlighted the importance of metabolic flexibility and redox balance in maintaining sperm viability and motility during *in vitro* storage and semen preservation procedures (4).

In this context, the preferential localization of AMSC-EV-associated fluorescence within the sperm midpiece is particularly interesting because this region contains the mitochondrial sheath responsible for ATP generation and sperm motility. Similar localization patterns were previously described in studies investigating EV-sperm interactions, suggesting that EVs may selectively interact with mitochondria-rich compartments of spermatozoa (14, 24). Although the precise molecular mechanisms remain unclear, the observed modulation of sperm kinetics suggests that AMSC-EV cargo may contribute to the regulation of membrane dynamics, intracellular signaling pathways, mitochondrial homeostasis, and sperm bioenergetic balance involved in motility persistence. Extracellular vesicles are known to transfer bioactive proteins, lipids, enzymes, and regulatory RNAs capable of influencing cellular metabolism and oxidative stress responses in recipient cells (6, 7).

Among the CASA-derived parameters, the increase in ALH observed after prolonged incubation deserves consideration. ALH reflects the lateral oscillation of the sperm head during movement and is commonly associated with changes in flagellar beating patterns and sperm trajectory dynamics. In the present study, AMSC-EV-treated spermatozoa initially showed lower ALH values together with higher LIN and STR values, indicating more organized and progressive movement during the early incubation phases. However, after the 4-h post-collection monitoring period, AMSC-EV-treated spermatozoa exhibited significantly increased ALH values compared with controls while still maintaining higher progressive motility and trajectory parameters. The late increase in ALH may reflect the emergence of a more vigorous flagellar beating pattern sharing some kinetic characteristics with sperm hyperactivation. Similar kinetic adaptations have previously been associated with modifications in calcium fluxes, mitochondrial activity, and membrane remodeling occurring during sperm functional activation (14). Nevertheless, the increase in ALH observed in the present study was not accompanied by evidence of premature acrosome reaction or loss of plasma membrane integrity in viable sperm cells, indicating that EV-induced kinetic changes did not appear to compromise sperm physiological integrity.

Extracellular vesicles are recognized as important mediators of reproductive tract communication and contribute to sperm maturation, capacitation, fertilization, and embryo–maternal signaling (14, 15). In recent years, MSC-EVs have gained increasing interest in regenerative medicine because they reproduce many of the anti-inflammatory and regenerative properties of their parental cells while avoiding several limitations associated with direct cell transplantation (6, 9, 10). Among the available MSC sources, amniotic tissue represents a particularly attractive origin for EV production due to its accessibility, low immunogenicity, and regenerative properties. Previous studies from our group demonstrated that equine AMSC-EVs possess anti-inflammatory and regenerative activity in reproductive tissues (12, 17, 18, 23). In addition, AMSC-EVs contain regulatory microRNAs and proteins involved in oxidative stress regulation and tissue repair (20, 22).

The beneficial effects observed in the present study are also consistent with previous reports showing that reproductive tract-derived EVs can modulate sperm physiology and improve fertilization-related processes (24). Similarly, amniotic fluid-derived exosomes improved spermatogenesis and testicular recovery in azoospermic rats, further supporting the regenerative potential of amniotic EVs within the male reproductive system (25). Together, these observations support the hypothesis that EV-mediated signaling may positively influence sperm functionality across different reproductive conditions.

One of the most relevant results of the present study is that the kinetic modulation induced by EV treatment was not associated with evidence of premature acrosome reaction in viable spermatozoa. Triple fluorescent staining demonstrated that acrosomal alterations mainly occurred in non-viable or degenerating sperm cells rather than as a direct consequence of AMSC-EV exposure. This observation is clinically relevant because maintenance of sperm physiological integrity is essential for practical application in assisted reproductive technologies. Previous studies investigating EV-mediated sperm modulation reported heterogeneous effects on capacitation and acrosomal status, likely due to differences in EV origin, species, incubation conditions, and EV cargo composition (14). Therefore, preservation of acrosomal stability in viable spermatozoa may represent an important advantage for future translational applications in stallion reproductive management.

The molecular mechanisms responsible for the observed effects remain speculative. Amniotic MSC-EVs contain proteins, lipids, antioxidants, and regulatory microRNAs potentially capable of modulating oxidative stress responses, membrane stability, and mitochondrial activity (20, 22). Previous studies identified anti-inflammatory and regulatory microRNAs within amniotic EV cargo, including miR-26a, miR-146a, and miR-335 (20), which may contribute to preservation of sperm functionality in aged stallions. However, additional proteomic, metabolomic, and functional studies will be necessary to identify the specific mediators responsible for the observed kinetic changes and to clarify whether the late increase in ALH reflects adaptive sperm activation, altered flagellar regulation, or changes in sperm energetic status during the 4-h post-collection monitoring period.

Another potentially relevant application concerns semen preservation and assisted reproductive technologies. Equine spermatozoa are particularly sensitive to cooling- and cryopreservation-induced mitochondrial damage, especially in aged stallions (3). Therefore, EV supplementation may represent a promising strategy for supporting semen handling and preservation protocols.

Recent evidence suggests that EV-based approaches may mitigate cryopreservation-associated sperm damage and improve post-thaw sperm functionality through the transfer of protective proteins, lipids, antioxidants, and regulatory RNAs (27).

These findings open a practical perspective for the use of AMSC-EVs as bioactive additives in stallion semen processing. A possible application could involve semen collection followed by short-term co-incubation with AMSC-EVs before cooled storage or cryopreservation, with the aim of enhancing sperm resilience to storage-associated stress and preserving post-cooling or post-thaw motility. Such an approach may be particularly valuable for aged stallions and poor cooler/freezer individuals. Nevertheless, dedicated studies are needed to determine whether EV-induced improvements in fresh semen are maintained after refrigeration or freezing and whether they translate into improved fertility outcomes.

Despite the encouraging results, several limitations should be acknowledged. First, only four stallions were enrolled and only one ejaculate per animal was analyzed. Therefore, despite considering each stallion as an independent biological replicate, the limited sample size reduces the statistical power and generalizability of the results. The study should consequently be considered a preliminary proof-of-concept investigation, and the results require confirmation in larger stallion populations and through the analysis of repeated ejaculates from each animal. Second, fertility outcomes and *in vivo* fertilizing ability were not evaluated. Finally, the molecular composition of EV cargo was not specifically investigated in relation to sperm functional improvement, preventing identification of the exact mediators involved.

In conclusion, this pilot study demonstrated that AMSC-EVs modulate sperm kinetic parameters in aged stallions during the 4-h post-collection monitoring period in aged stallions while preserving sperm physiological integrity and acrosomal stability in viable spermatozoa. To the authors’ knowledge, this is the first study demonstrating the effects of AMSC-EVs on sperm kinetics in aged stallions. These findings support the concept that modulation of sperm bioenergetics may represent a promising therapeutic target for age-related subfertility in stallions. Further studies are warranted to clarify the molecular mechanisms involved and evaluate the translational applicability of EV-based strategies in equine reproductive medicine.

## Data Availability

The datasets presented in this study can be found in online repositories. The names of the repository/repositories and accession number(s) can be found in the article/ Supplementary material.
